# Growing Literature, Stagnant Science? Systematic Review, Meta-Regression and Cumulative Analysis of Audit and Feedback Interventions in Health Care

**DOI:** 10.1007/s11606-014-2913-y

**Published:** 2014-06-26

**Authors:** Noah M. Ivers, Jeremy M. Grimshaw, Gro Jamtvedt, Signe Flottorp, Mary Ann O’Brien, Simon D. French, Jane Young, Jan Odgaard-Jensen

**Affiliations:** 1 Family Practice Health Centre and Institute for Health Systems Solutions and Virtual Care, Women’s College Hospital, Toronto, Ontario Canada; 2 Clinical Epidemiology Program, Ottawa Hospital Research Institute, Department of Medicine, University of Ottawa, 725 Parkdale Ave., Ottawa, Ontario K1Y 4E9 Canada; 3 Norwegian Knowledge Centre for the Health Services, 7004 St. Olavs Plass, 0130 Oslo, Norway; 4 School of Rehabilitation Therapy, Faculty of Health Sciences, Queen’s University, 31 George Street, Room 222, Kingston, Ontario K7L 3N6 Canada; 5 Cancer Epidemiology and Services Research, Sydney School of Public Health, University of Sydney, Sydney, New South Wales 2006 Australia

**Keywords:** audit and feedback, scientific progress, quality improvement, systematic review, cumulative analysis

## Abstract

**BACKGROUND:**

This paper extends the findings of the Cochrane systematic review of audit and feedback on professional practice to explore the estimate of effect over time and examine whether new trials have added to knowledge regarding how optimize the effectiveness of audit and feedback.

**METHODS:**

We searched the Cochrane Central Register of Controlled Trials, MEDLINE, and EMBASE for randomized trials of audit and feedback compared to usual care, with objectively measured outcomes assessing compliance with intended professional practice. Two reviewers independently screened articles and abstracted variables related to the intervention, the context, and trial methodology. The median absolute risk difference in compliance with intended professional practice was determined for each study, and adjusted for baseline performance. The effect size across studies was recalculated as studies were added to the cumulative analysis. Meta-regressions were conducted for studies published up to 2002, 2006, and 2010 in which characteristics of the intervention, the recipients, and trial risk of bias were tested as predictors of effect size.

**RESULTS:**

Of the 140 randomized clinical trials (RCTs) included in the Cochrane review, 98 comparisons from 62 studies met the criteria for inclusion. The cumulative analysis indicated that the effect size became stable in 2003 after 51 comparisons from 30 trials. Cumulative meta-regressions suggested new trials are contributing little further information regarding the impact of common effect modifiers. Feedback appears most effective when: delivered by a supervisor or respected colleague; presented frequently; featuring both specific goals and action-plans; aiming to decrease the targeted behavior; baseline performance is lower; and recipients are non-physicians.

**DISCUSSION:**

There is substantial evidence that audit and feedback can effectively improve quality of care, but little evidence of progress in the field. There are opportunity costs for patients, providers, and health care systems when investigators test quality improvement interventions that do not build upon, or contribute toward, extant knowledge.

## BACKGROUND

Audit and feedback is widely used as a strategy to improve professional practice, either on its own or as a key component of multifaceted quality improvement (QI) interventions. Providing data regarding clinical performance may overcome health professionals’ limited abilities to accurately self-assess their performance.^1^ It is posited that when well-designed feedback demonstrates suboptimal performance for important and actionable targets, recipients are more likely to respond with efforts to improve quality of care.^2^

**Figure 1:**
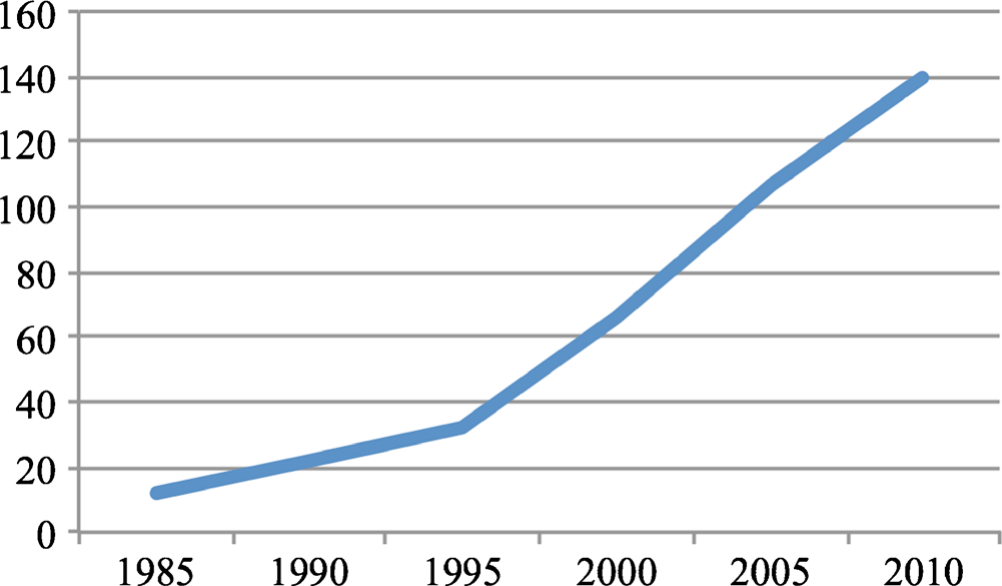
Cumulative number of randomized trials featuring audit and feedback as a core component of a quality improvement intervention.

The most recent Cochrane systematic review and meta-analysis of audit and feedback included 140 randomized clinical trials (RCTs),^3^ making audit and feedback one of the most studied healthcare quality improvement (QI) interventions. Three Cochrane reviews over the course of 10 years came to the same conclusion: Audit and feedback generally leads to small but potentially important improvements in professional practice (Table 1).^3^^–^ 5 Yet despite the increasing number of audit and feedback trials (Fig. 1), uncertainty remains regarding when audit and feedback is likely to be most helpful and how to best optimize the intervention.^6^

**Table 1:** Findings from Cochrane Systematic Reviews and Meta-Analyses of Audit and Feedback Over Time

Year of review	Effect size	Conclusion
2003 (search up to January 2001)	Forty-seven studies with dichotomous outcomes: 7 % (IQR: 2–11) median absolute increase in compliance with intended professional behaviors or processes	“Audit and feedback can be effective in improving professional practice. When it is effective, the effects are generally small to moderate. The absolute effects of audit and feedback are more likely to be larger when baseline adherence to recommended practice is low.”^4^
2006 (search up to January 2004)	Forty-nine studies with dichotomous outcomes: 5 % (IQR: 3–11) median absolute increase in compliance with intended professional behaviors or processes	“Audit and feedback can be effective in improving professional practice. The effects are generally small to moderate. The absolute effects are likely to be larger when baseline adherence to recommended practice is low and intensity of audit and feedback is high.”^5^
2012 (search up to December 2010)	Sixty-two studies with dichotomous outcomes: 4 % (IQR: 1–16) weighted median absolute increase in compliance with intended professional behaviors or processes	“Audit and feedback generally leads to small but potentially important improvements in professional practice. The effectiveness of audit and feedback seems to depend on baseline performance and how the feedback is provided. Future studies of audit and feedback should directly compare different ways of providing feedback.”^3^

*IQR* interquartile range

In some instances, audit and feedback is highly effective; learning from such examples is necessary to optimize the effectiveness of the intervention across different contexts. The Cochrane review and associated re-analyses have found that the effectiveness of audit and feedback depends to some extent on how the intervention is designed and delivered, suggesting an opportunity to maximize the impact of this QI strategy on quality of care.^3^^,^ 7 ^,^ 8 However, there is evidence that many audit and feedback interventions are developed and tested without an explicit attempt to consider relevant theories or to build upon extant knowledge.^9^ Ideally, results of early studies would inform the design of future interventions, and through this process, cumulative knowledge would lead to more effective QI. Given the continuing human and financial capital invested in audit and feedback interventions in health care, it is important to examine whether newer trials of audit and feedback have contributed new knowledge to the field.

The purpose of this paper is to extend the results of the Cochrane review of audit and feedback to explore the evolution of evidence supporting this QI intervention over time. In particular, we examined whether effect estimates, and the precision around those estimates, changed over time. To do this, we undertook a cumulative analysis of trials by year of publication and conducted a series of meta-regressions to understand how the literature has developed with respect to determining factors that could explain why audit and feedback is more or less effective.

## METHODS

This is a secondary analysis of data from the previously published Cochrane systematic review of audit and feedback. Complete methodological details are available^3^ and are summarized below. Ethics approval was not required for this study.

### Eligibility Criteria

Audit and feedback was defined as a “summary of clinical performance of health care over a specified period of time.” This secondary analysis only included RCTs that directly compared audit and feedback (either alone or as the core, essential feature of a multifaceted intervention) to usual care. Furthermore, only RCTs that evaluated effects on provider practice as a primary outcome were included. For ease of interpretation of the meta-regression and cumulative meta-analysis, we further limited studies to those that reported dichotomous outcomes (i.e., compliance with intended professional practice).

### Information Sources, Search, and Study Selection

A search strategy sensitive for RCTs involving audit and feedback was applied in December 2010 to the Cochrane Central Register of Controlled Trials, MEDLINE, EMBASE, and CINAHL. As previously described,^3^ we developed a MEDLINE search strategy that identified 89 % of all MEDLINE indexed studies from the previous version of the review and then translated this strategy into the other databases using the appropriate controlled vocabulary as applicable. Search terms included: audit, benchmarking, feedback, utilization review, health care quality, etcetera, plus typical search terms to focus on RCTs. Two reviewers independently screened the titles, abstracts, and full texts to apply inclusion criteria.

### Data Collection Process

Two reviewers independently abstracted data from included studies. Studies included in the previous version of the Cochrane review of audit and feedback were reassessed due to changes in the data abstraction form and methods. Discrepancies were resolved through discussion. For studies lacking extractable data or without baseline information, we contacted investigators via email. Risk of bias for the primary outcome(s) in each study was assessed according to the Cochrane Effective Practice and Organization of Care group criteria^10^ (sequence generation, allocation concealment, blinding, incomplete outcome data, selective reporting, baseline similarity, lack of contamination, and other). We assigned an overall assessment of the risk of bias for each study as high, moderate, or low, following the recommendations in the Cochrane Handbook.^11^ Studies with a high risk of bias in at least one domain that decreased the certainty of the effect size of the primary outcome were considered to have a high risk of bias. Conversely, when a study had low risk of bias for each domain, it was deemed low risk of bias overall. Other studies were considered to have unclear risk of bias.

### Measure of Treatment Effect

We only extracted results for the primary outcome. When the primary outcome was not specified, we used the variable described in the sample size calculation as the primary outcome. When the primary outcome was still unclear or when the manuscript described several primary process outcomes, we calculated the median value. We calculated the treatment effect as an adjusted risk difference (RD) by subtracting baseline differences from post-intervention differences. Thus, an adjusted RD of +10 % indicates that after accounting for baseline differences, health professionals receiving the intervention adhered to the desired practice 10 % more often than those not receiving the intervention.

### Analysis

Across multiple studies, we weighted the median effect by the number of health care providers. The ‘median of medians’ technique has been used in many similar reviews evaluating the effect of QI interventions on health professional performance,^12^ due to frequency of unit of analysis errors in the literature and the great variety of clinical contexts covered in the studies. For the cumulative analysis, the median adjusted RD and interquartile range (IQR) was recalculated at each time point as studies were added. The meta-regression examined how the adjusted RD was related to explanatory variables, weighted according to study size (number of health care professionals). Unlike the meta-regression from the Cochrane review of audit and feedback,^3^ high risk of bias studies were included. The meta-regression also tested the following potential sources of heterogeneity to explain variation in the results of the included studies: format (verbal, written, both, unclear); source (supervisor or senior colleague, professional standards review organization or representative of employer/purchaser, investigators, unclear); frequency (weekly, monthly, less than monthly, one-time); instruction for improvement (explicit measurable target or specific goal but no action plan, action plan with suggestions or advice given to help participants improve but no goal/target, both, neither); direction of change required (increase current behavior, decrease current behavior, mix or unclear); recipient (physician, other health professional); and study risk of bias (high, unclear, low). Meta-regression was conducted for all published trials as of 2010, 2006 and 2002. Finally, we added year of publication as a continuous variable to the meta-regression of all studies as an additional approach to assess whether this variable accounted for a significant portion of the heterogeneity. We conducted a multivariable linear regression using main effects only. Baseline compliance and year of publication were treated as continuous explanatory variables and the others as categorical. The analyses were conducted using the GLIMMIX procedure in SAS Version 9.2 (SAS Institute Inc. Cary, NC USA), accounting for the dependency between comparisons from the same trial.

## RESULTS

**Figure 2:**
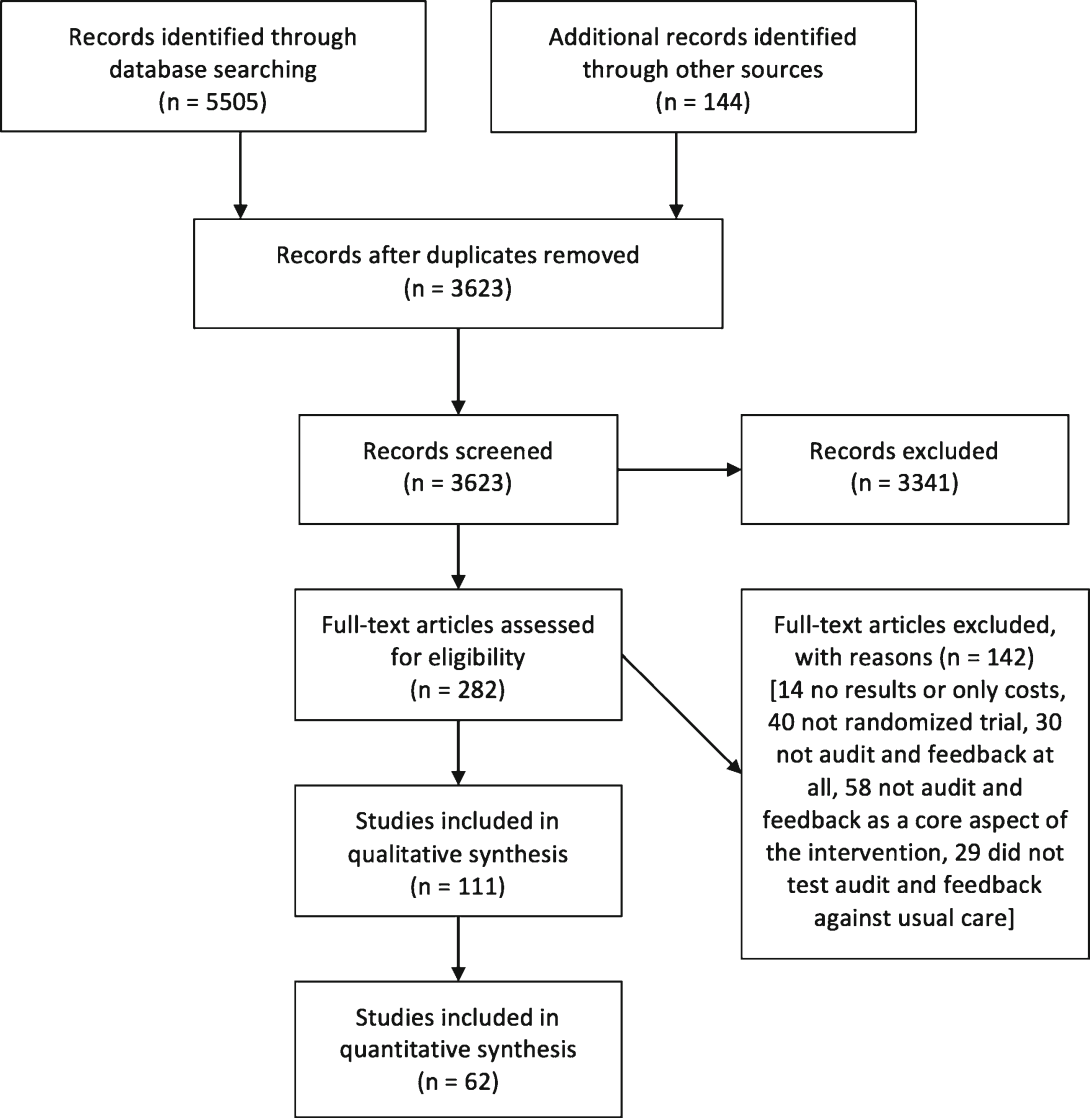
Study flow diagram.

Of the 140 RCTs included in the Cochrane review, 98 comparisons from 62 studies met the criteria for this study (Fig. 2). These studies included over 2,300 groups of healthcare providers (e.g., clinics or hospitals) from 38 trials allocating clusters of professionals, and more than 2,000 professionals from 24 trials allocating individual healthcare providers. Characteristics of these studies are described in Table 2. Most studies took place in the USA or Canada (55 %), and outpatient care was the most common setting (69 %). The feedback was delivered by either the investigators or by an unclear source in 85 % of studies. In 47 % of the studies, the feedback was only delivered once, and in 61 % the feedback did not include an explicit goal or action plan.

**Table 2:** Characteristics of Studies

Study characteristic	No.	%	Intervention characteristic	No.	%
Publication year			Format		
2007–2009	10	16.1	Verbal	6	9.7
2003–2006	22	35.5	Written	34	54.8
1996–2002	20	32.4	Both	18	29.0
1986–1995	5	8.1	Unclear	4	6.5
Before 1986	5	8.1	Source		
Country			Supervisor/colleague	6	9.7
USA	27	43.6	Employer	3	4.8
UK or Ireland	7	11.3	Investigators/unclear	53	85.5
Canada	7	11.3	Frequency		
Australia or New Zealand	6	9.7	Monthly or more	11	17.7
Other	15	24.2	Repeated less than monthly	19	30.7
Unit of allocation			Once only	29	46.8
Provider	24	38.7	Instructions for improvement		
Many providers/groups	38	61.3	Goal-setting	5	8.1
Unit of analysis			Action planning	16	25.8
Patient	37	59.7	Both	3	4.8
Provider	12	19.4	Neither	38	61.3
Many providers/groups	12	19.4	Nature of change required		
Unclear	1	1.6	Increase current behavior	29	46.8
Risk of bias			Decrease current behavior	6	9.7
Low	21	33.9	Mix or unclear	27	43.6
Unclear	29	46.8	Clinical topic		
High	12	19.4	Diabetes	11	17.7
Number of arms in trial			Laboratory testing/radiology	3	4.8
Two	41	66.1	Prescribing	18	29.0
Three	10	16.1	Other	30	48.4
Four	11	17.7	Targeted health professional		
Clinical setting			Physicians	51	82.3
Outpatient	43	69.4	Other	11	17.7
Inpatient	14	22.6	Medical specialty (could be > 1)		
Other/unclear	5	8.1	GP/family physician	39	62.9
			Internists	25	40.3
			Other	20	32.3

**Figure 3:**
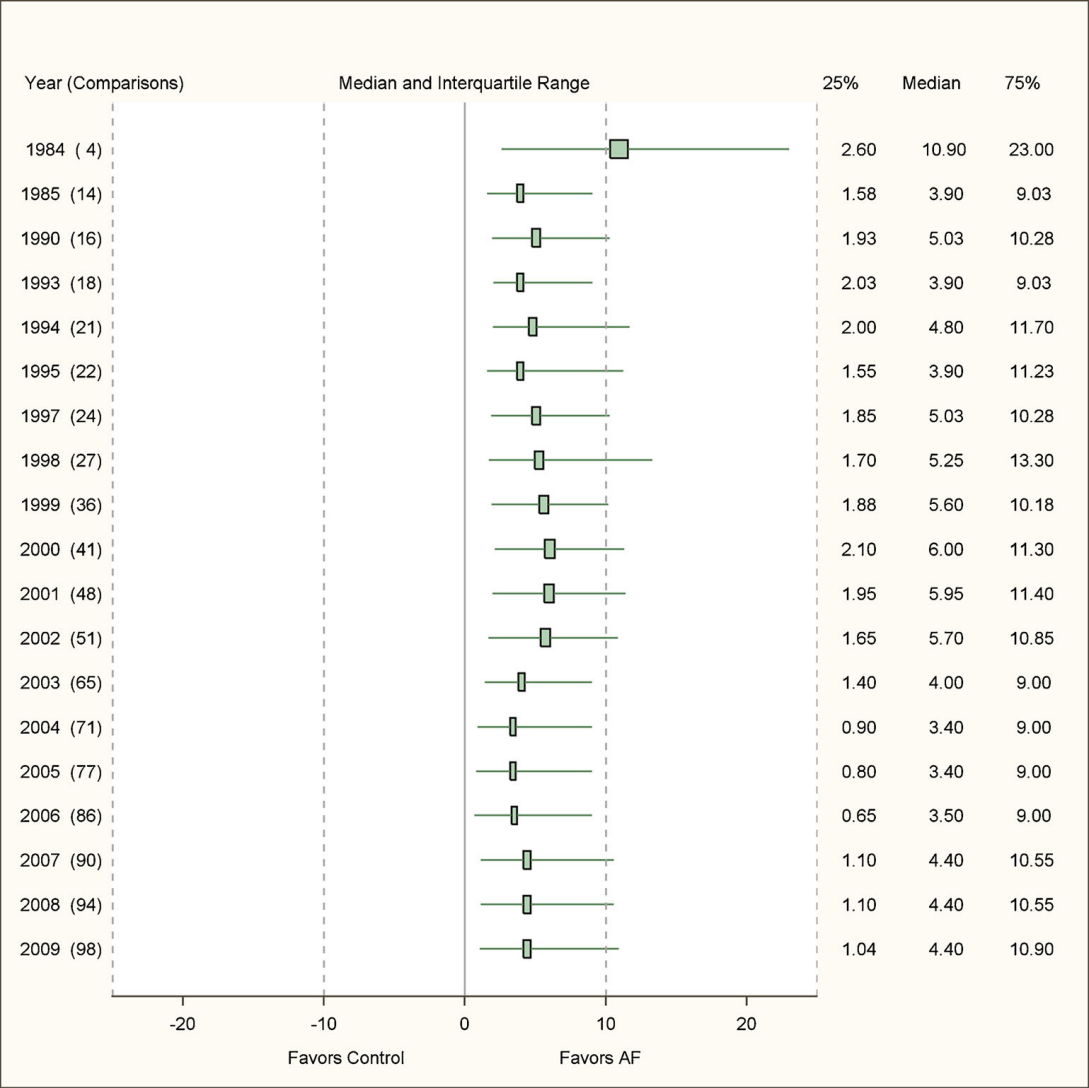
Cumulative analysis–effect size* of audit and feedback interventions over time (AF: audit and feedback; *absolute difference in compliance with intended professional behaviors).

The cumulative analysis revealed little change in the median effect or the interquartile range over the course of 25 years (Fig. 3). The median improvement in adherence to intended practice in 2002 after 51 comparisons had been published was 5.7 (IQR = 1.65–10.85), the effect in 2006 after 86 comparisons was 3.5 (IQR = 0.65–9.00), and the effect after including all 98 comparisons was 4.4 (IQR = 1.04–10.90).

The meta-regression revealed that heterogeneity in effect sizes could be explained in part by feedback characteristics, but year of publication did not explain a significant portion of the variability in effect size (Table 3). Feedback seemed most effective when it was: delivered from a supervisor or respected colleague; presented more than once; featuring both specific goals and action-plans; aiming to decrease the targeted behavior; with lower baseline performance; and when recipients were non-physicians. Studies published after 2006 did not change the meta-regression results statistically; differences in the estimated effect for most feedback characteristics have been apparent qualitatively since 2002. For example, although the p value was not significant for source of feedback in 2002, the estimated adjusted risk difference for feedback delivered by a supervisor or respected colleague (24.5) was higher than feedback delivered by study investigators (17.9) or feedback from a representative of a regulatory agency or employer (0.9).

**Table 3:** Factors Explaining Variability in Effectiveness of Feedback: Serial Meta-Regressions

Characteristic of feedback	Estimated effect size^*^, (no. studies)		
	2010	2006	2002
Format of feedback	*p* = 0.386	*p* = 0.731	*p* = 0.729
Verbal	12.77, (15)	14.85, (14)	17.02, (12)
Written	20.70, (50)	19.94, (41)	23.76, (19)
Both verbal and written	19.05, (27)	19.19, (26)	16.98, (18)
Not clear	16.90, (6)	13.58, (5)	2.94, (2)
Source of feedback	*p* = 0.006	*p* = 0.034	*p* = 0.300
A supervisor or respected colleague	25.22, (10)	23.49, (8)	24.48, (4)
Standards review org. or representative of employer	9.16, (3)	9.38, (3)	0.90, (1)
The investigators	15.19, (52)	14.71, (42)	17.85, (13)
Not clear	19.85, (33)	19.99, (33)	17.47, (33)
Frequency of feedback	*p* < 0.001	*p* < 0.001	*p* < 0.001
Frequent (up to weekly)	27.58, (5)	28.50, (3)	28.64, (2)
Moderate (up to monthly)	18.51, (10)	16.73, (9)	18.31, (4)
Infrequent (less than monthly)	14.04, (26)	13.32, (22)	1.06, (10)
Once only	7.49, (52)	7.75, (47)	9.96, (30)
Unclear;	19.15, (5)	18.17, (5)	17.92, (5)
Instructions for improvement	*p* = 0.044	*p* = 0.068	*p* = 0.325
Explicit, measurable target, but no action plan	10.88, (5)	10.45, (5)	8.48, (1)
Action plan, but no explicit target	17.16, (32)	16.69, (31)	11.37, (18)
Both	23.19, (4)	23.06, (4)	22.01, (4)
Neither;	18.18, (57)	17.37, (46)	18.84, (28)
Nature of change required	*p* = 0.025	*p* = 0.028	*p* = 0.510
Increase current behavior	15.55, (40)	15.65, (36)	19.34, (17)
Decrease current behavior	22.46, (11)	22.30, (11)	12.61, (4)
Change behavior to similar alternative or unclear	14.05, (47)	12.73, (39)	13.58, (30)
Profession of recipient (Physician yes/no)	*p* < 0.001	*p* < 0.001	*p* < 0.001
Physician	10.99, (82)	10.19, (72)	4.80, (45)
Not physician	23.72, (16)	23.60, (14)	25.55, (6)
Risk of bias	*p* = 0.375	*p* = 0.564	*p* = 0.281
Yes (low risk of bias)	14.85, (32)	14.92, (27)	21.34, (8)
Unclear	15.79, (51)	15.33, (48)	10.06, (34)
No (high risk of bias);	21.42, (15)	20.43, (11)	14.12, (9)
Baseline performance (continuous variable)	*p* < 0.001	*p* = 0.003	*p* = 0.021

^*^Absolute difference in compliance with intended professional behaviors

## DISCUSSION

Audit and feedback works; the median effect is small though still potentially important at the population level, and 27/98 comparisons (28 %) resulted in an improvement of at least 10 % in quality of care.^3^ Small differences in the results seen in these re-analyses compared to the results of the Cochrane review are due to the lack of weighting in the cumulative analysis and the inclusion of high risk of bias studies in the meta-regression. Nevertheless, the expected effect of an intervention comparing audit and feedback to usual care has changed very little over the last two decades. Furthermore, new trials have provided little new knowledge regarding key effect modifiers. Given the lack of equipoise, it may no longer be ethically appropriate to continue to direct human and financial resources toward trials comparing audit and feedback against usual care, especially for common conditions in common settings. At this point, the appropriate question is not, ‘can audit and feedback improve professional practice?’ but ‘how can the effect of audit and feedback interventions be optimized?’

Based on our analyses, feedback seems most effective when it: is delivered by a supervisor or respected colleague; is presented frequently; includes both specific goals and action-plans; aims to decrease the targeted behavior; focuses on a problem where there was larger scope for improvement; and when the recipients are non-physicians. Unfortunately, relatively few trials feature these components. Furthermore, our findings suggest that investigators are not building upon best practices. For example, despite evidence that repeated feedback is more effective, studies that evaluate interventions after only one cycle of feedback continue to be performed. Furthermore, of the 32 studies conducted after 2002 considered in this analysis, feedback was delivered by a supervisor or respected colleague only six times, and no studies included feedback with both explicit goals and action plans. As a result, even after 140 randomized trials of audit and feedback, it remains difficult to identify how to optimize audit and feedback.^6^ For instance, although a ‘supervisor or respected colleague’ appears to be the most effective source to deliver feedback, precise strategies to reliably identify and leverage such sources are not well known.^13^ In addition, while it is advisable for action plans to accompany feedback since the downside is minimal, the best way to operationalize this is unknown.^7^^,^ 14 It is noteworthy that explicit targets without action plans do not seem to be particularly helpful. To achieve performance targets, recipients of feedback benefit from correct solution information^8^ that can focus their attention on the targeted behavior(s).

Cumulative meta-analyses have previously been used to investigate whether future trials would be likely to change the conclusions regarding the effectiveness of QI or health services interventions.^15^^,^ 16 For audit and feedback, it is plausible that further studies comparing the intervention against control may be informative if they are conducted for settings, professional groups or behaviors not well targeted in the current review (although relatively few additional trials should be needed to confirm whether observed effects are broadly aligned with observed effects across the body of literature). We recognize the risks of cumulative meta-analysis with respect to multiple testing and escalating type one error.^17^ However, since the Cochrane review did not include a variance around the intervention effect, the figures showing the results of our cumulative analysis do not feature error bars as in the seminal examples of Lau et al.^18^ Additionally, the number of characteristics tested in the meta-regression was limited by statistical and pragmatic concerns. Variables were only chosen for abstraction if there was an a priori directional hypothesis and a belief that data would be available in published reports. Confidence in the results of the meta-regression is limited by reliance upon indirect comparisons and risk of ecological fallacy. In other words, relationships identified across studies through meta-regression may not reflect relationships evident within studies; this is also known as aggregation bias. Finally, as with any review, the limitations of the primary studies must be considered.

We acknowledge that many other potential variables, including the clinical topic and context, likely impact the effectiveness of the intervention.^19^^,^ 20 Amongst the 98 comparisons, there were 41 comparisons testing audit and feedback alone and 57 comparisons testing audit and feedback as the core, essential part of a multifaceted intervention. It is plausible that co-interventions may interact with the effect modifiers tested in the meta-regressions. A recent international meeting was conducted to identify high-yield research questions for understanding how to enhance the effectiveness of audit and feedback. Stakeholders suggested a need for more research to better understand how contextual and recipient characteristics moderate audit and feedback effectiveness, characteristics of the desired behavior change that make a good target for audit and feedback, and how the specific design of the audit and feedback intervention interacts with these factors.^21^

Given the importance of audit and feedback as a key component of many QI interventions, there is a need to identify opportunities to sequentially and systematically test various approaches to the design and development of audit and feedback. Researchers can continue to conduct uncoordinated trials of audit and feedback versus usual care and rely upon periodically conducted meta-regressions across studies to explore effect modifiers. But the results will be at risk of ecological fallacies, and as demonstrated here, this approach has resulted in minimal advances over time. Alternatively, researchers could achieve greater confidence in causal inference regarding more effective intervention design through a limited number of multi-arm trials with direct, head-to-head comparisons testing different approaches for designing and delivering audit and feedback. Another approach that could help advance cumulative knowledge regarding audit and feedback and other QI strategies would be to consider engineering-based methodological options that enable testing of multiple potential effect modifiers, such as theory-driven factorial and/or sequential adaptive trials.^22^ Future audit and feedback interventions should feature the aspects known to be associated with greater effectiveness and future trials should be powered to find relatively small effect sizes, especially in the case of head-to-head trials. This proposed shift in direction for QI trials parallels the movement to limit placebo-controlled trials of clinical interventions and to increase focus on comparative effectiveness research.^23^

The findings of this review suggest that QI trialists have failed to cumulatively learn from previous studies (or from systematic reviews). Rather, it would appear that the norm for those testing audit and feedback interventions is to ‘re-invent the wheel’, repeating rather than learning from and contributing to extant knowledge.^24^ As highlighted in the recent series on increasing value and reducing waste in research,^25^ the opportunity cost of continuing in the current manner is large for patients, providers, and health systems. A coordinated approach toward building upon previous literature and relevant theory to identify the key, active ingredients of interventions would help QI stakeholders achieve greater impact with their interventions and produce outcomes that are more generalizable.^26^^,^ 27 In particular, QI trialists could benefit from adapting the model of the Children’s Oncology Group, which has successfully shared resources to accelerate progress.^28^ At a minimum, for stakeholders involved in the funding and conduct of QI trials, this analysis emphasizes the need for trials of carefully planned interventions with explicitly justified components to ensure that the field of QI in healthcare can move forward.
